# Insight into the antiviral activity of synthesized schizonepetin derivatives: A theoretical investigation

**DOI:** 10.1038/s41598-020-65866-5

**Published:** 2020-05-25

**Authors:** Alireza Baghban, Amir Mosavi

**Affiliations:** 1 Department of chemical engineering, Amirkabir University of Technology, Mahshahr campus, Mahshahr, Iran; 2 Institute of Research and Development, Duy Tan University, Da Nang, 550000 Vietnam; 3 Department of Mathematics, J. Selye University, 94501 Komarno, Slovakia; 4 Kalman Kando Faculty of Electrical Engineering, Obuda University, 1034 Budapest, Hungary

**Keywords:** Drug discovery, Nanoscience and technology

## Abstract

The antiviral activity of schizonepetin derivatives 1A-1C were investigated via theoretical methods and results are compared with experimental results. The derivatives 1 A and 1 C have the highest and the lowest antiviral activity, respectively. The interactions of derivatives 1A-1C and BN-nanotube are examined. Results show that, derivatives 1A-1C can effectively interact with BN-nanotube (9, 9) and their adsorptions are favorable. The energy of derivative 1 A is higher than derivatives 1B and 1 C. The derivative 1 A has highest absolute µ, ω and ∆N values and it has lowest absolute ƞ value. Results show that, theoretical and experimental trends of antiviral activity of derivatives 1A-1C were similar, successfully.

## Introduction

The schizonepetin structures (1A-1C) were synthesized and their antiviral activities are studied. The antiviral potential of schizonepetin structures (1A-1C) against HSV-1 and influenza H3N2 were investigated in Table 1^1–6^.

**Table 1:** Structure of schizonepetin derivatives 1A-1C.

Derivatives	Group	HSV-1 virus	H_3_N_2_ virus
1A	F	77.8	60.5
1B	Br	80.3	71.0
1C	CF_3_	82.8	75.4
1A			
1B			
1C			

The derivative 1 A is the most active drug against HSV-1 virus and influenza virus H3N2. Derivative 1 C has higher TAC_50_ values and so has lowest activity HSV-1 and influenza. The structure analysis of derivative 1A-1C shown that the F, Br and CF_3_ substituents have high important role in antiviral activity of synthesized schizonepetin. The F and Br atoms of derivatives 1 A and 1B can share their electrons pairs to resonate with unsaturated ring and they have high potential to stable the schizonepetin and these structures can have high potential to adsorb the electrons. About derivative 1 C the CF_3_ group is reduced he stability of schizonepetin and it cannot share electrons with unsaturated ring, therefore derivative 1 C has lower activity than derivatives 1 A and 1B. Results indicate that antiviral activity of schizonepetin derivatives 1A-1C in according to TAC_50_ scale decreased in the following order: 1 C < 1B < 1 A^1,7–12^.

**Figure 1:**
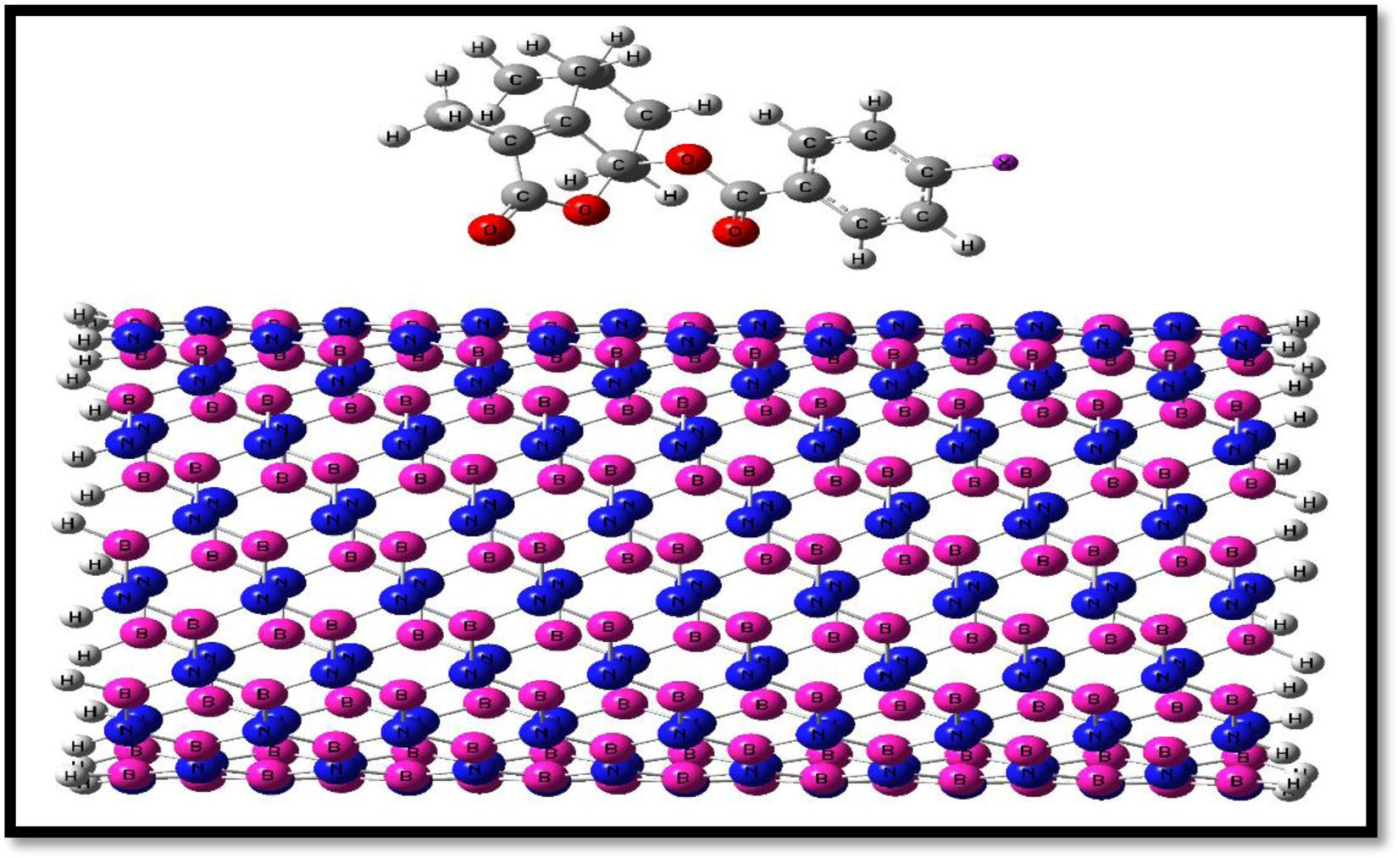
Structure of complexes of schizonepetin derivatives 1A-1C with BN-nanotube (9, 9).

In the present work, the antiviral potential of synthesized schizonepetin derivatives 1A-1C (structures were shown in Table 1) are studied. In this study the µ, ƞ, ω and ∆N related to schizonepetin derivatives 1A-1C and BN-nanotube (9, 9) were investigated. The energies of derivatives 1A-1C and nanotubes were examined (Fig. 1). These results can be useful to predication the potential of nanotube to derivatives 1A-1C based on calculated quantum molecular descriptors^2,13–16^.

The aims are: (1) to calculate the antiviral potential of schizonepetin derivatives 1A-1C; (2); to find derivatives 1A-1C with higher antiviral activity; (3) to compare the ∆E_ad_ and ∆G_ad_ of derivatives 1A-1C on BN-nanotube surface; (4) to investigate the quantum molecular descriptors of derivatives 1A-1C; (5) to compare the theoretical and experimental trends of antiviral activity of derivatives 1A-1C.

## Computational details

The structures of schizonepetin derivatives 1A-1C are optimized by DFT/B3LYP and 6–31 G (d, p). The adsorption energy of schizonepetin derivatives 1A-1C on BN-nanotube (9, 9) surface is ∆E_ad_ = E (BN-nanotube (9, 9)/drug) – E (drug) – E (BN-nanotube (9, 9)) + E_BSSE_. The negative ∆E_ad_ and ∆G_ad_ shown that the adsorption of derivatives 1A-1C on BN-nanotube (9, 9) are favorable reaction^14,15,17–19^.

## Results and discussion

### Calculated ∆E_ad_ and ∆G_ad_ of schizonepetin derivatives 1A-1C on nanotube

The F, Br and CF_3_ synthesized derivatives of schizonepetin have high antiviral activity than other derivatives. The experimental researchers confirmed that F, Br and CF_3_ synthesized derivatives of schizonepetin can be synthesized more comfortable than other derivatives. The experimental researchers shown that F, Br and CF_3_ synthesized derivatives of schizonepetin have most antiviral active against HSV-1 virus and influenza virus H3N2 ^20–25^.

The ∆E_ad_ and ∆G_ad_ of schizonepetin derivatives 1A-1C on nanotubes are stated in Table 2. The ∆E_ad_ and ∆G_ad_ are negative and the adsorption of derivatives 1A-1C on studied BN-nanotube (9, 9) are favorable processes. The ∆E_ad_ of derivatives 1 A and 1B are higher than derivative 1 C. The ∆G_ad_ of derivatives 1 A on BN-nanotube (9, 9) are higher than derivatives 1B and 1 C ca 0.10 and 0.17 eV. The ∆G_ad_ value of derivative 1B on BN-nanotube (9, 9) are more negative than derivative 1 C ca 0.07 eV. The derivative 1 A has the best ability to nanotube adsorption. These results can be interpret based on this fact that the electrons of orbitals of F and Br groups have higher interactions with unoccupied orbitals of BN-nanotube (9, 9). The electrons of C atoms of CF_3_ group have lower potential to interaction with orbitals of BN-nanotube (9, 9). Therefore, the ∆E_ad_ and ∆G_ad_ of derivatives 1 A and 1B are more negative than derivative 1 C and the most interactions are obtained for derivatives 1 A and BN-nanotube (9, 9).

**Table 2:** Calculated ∆E_ad_ and ∆G_ad_ (in eV) of schizonepetin derivatives 1A-1C on BN-nanotube (9, 9) surface.

Structures	∆E_ad_	∆G_ad_
1A	−0.54	−0.45
1B	−0.42	−0.35
1C	−0.36	−0.28

### Calculated quantum molecular descriptors of schizonepetin derivatives 1A-1C and BN-nanotube (9, 9)

The calculated energy parameters for schizonepetin derivatives 1A-1C and BN-nanotube (9, 9) are reported in Table 3. The calculated µ value of BN-nanotube (9, 9) is −0.56 eV. The calculated µ value of derivatives 1A-1C ranges from −0.45 to −0.47 eV and absolute µ values of them decreases in the order: 1 A > 1B > 1 C. Therefore, obtained absolute µ values show that derivative 1 A has highest electron and derivative 1 C has lowest electron.

**Table 3:** Calculated µ, η, ω and ∆N (in eV) of schizonepetin derivatives 1A-1C and BN-nanotube (9, 9).

Structures	µ	η	ω	∆N
BN-nanotube (9, 9)	−0.56	0.09	1.81	—
1 A	−0.47	0.08	1.44	−0.281
1B	−0.46	0.12	0.88	−0.232
1 C	−0.45	0.17	0.60	−0.220

In Table 3, the ƞ of BN-nanotube (9, 9) is 0.09 eV. The obtained ƞ values of derivatives 1A-1C decrease in the order: 1 A < 1B < 1 C. As the minimum of the ƞ value within the derivatives 1A-1C is for derivative 1 A. Therefore, ƞ values show that 1 A has lowest stability and high reactivity and 1 C has lowest reactivity. These results can be interpret based on this fact that the F and Br atoms of derivatives 1 A and 1B are shared electrons to unsaturated ring and they have high potential to stable the schizonepetin. In the derivative 1 C the CF_3_ substituent can decrease the stability of schizonepetin and C atoms of CF_3_ do not transfer the electrons to ring of schizonepetin. Therefore, it can be concluded the derivative 1 C has lower activity than derivatives 1 A and 1B.

Calculated ω value of BN-nanotube (9, 9) is 1.81 eV. The calculated ω value of derivatives 1A-1C ranges from 0.60 to 1.44 eV. Among the derivatives 1A-1C the ω value decreases in the order: 1 A > 1B > 1 C. Therefore, obtained ω values show that derivative 1 A has highest capacity to accept electrons and derivative 1 C has lowest capacity to accept electrons.

The calculated ∆N value of complexes of derivatives 1A-1C with BN-nanotube (9, 9) are reported in Table 3. The all of the calculated ∆N values are negative and derivatives 1A-1C can act as electron donors and BN- nanotube (9, 9) can act as electron acceptors. Results show that derivative 1 A has highest absolute ∆N value and it has highest interaction with BN-nanotube (9, 9). The derivative 1 C has lowest absolute ∆N value and it has lowest interaction with BN-nanotube (9, 9).

### Comparison of experimental and theoretical trends of antiviral activity of schizonepetin derivatives 1A-1C

The antiviral activity of derivatives is decreased as follow: 1 C < 1B < 1 A. The adsorption ability of derivatives 1A-1C via adsorption parameters (∆E_ad_ and ∆G_ad_) is: 1 A > 1B > 1 C. The obtained µ, ƞ and ω values show that derivative 1 A has highest absolute µ and ω values and it has lowest absolute ƞ values. Also derivative 1 C has lowest absolute µ and ω values and it has highest ƞ value.

This can be concluded the calculated µ, ƞ, ω values of derivatives 1A-1C in section 3.3 and energies is same. The highest absolute ∆E_ad_, ∆G_ad_, µ and ω values and lowest ƞ value for derivative 1 A are appropriate benchmark to approval the adsorption ability on BN-nanotube (9, 9) surface. The ∆E_ad_, ∆G_ad_, µ, ƞ, ω values of schizonepetin derivatives 1A-1C can consider as important parameters to predicate the adsorption ability on BN-nanotube (9, 9) surface.

## Conclusion

In this study, the antiviral activity of schizonepetin derivatives 1A-1C are investigated via theoretical methods. The derivatives 1 A and 1 C have the highest and the lowest of antiviral activity, respectively. The interactions of derivatives 1A-1C with BN-nanotube (9, 9) are investigated and also quantum molecular descriptors of derivatives 1A-1C are calculated. The energies of derivatives 1A-1C on BN-nanotube (9, 9) surface are studied. The adsorption ability of derivatives 1A-1C in according to adsorption parameters is: 1 A > 1B > 1 C. The derivative 1 A has the highest absolute µ and ω values and it has the lowest absolute ƞ value. Results show that, quantum molecular descriptors and adsorption parameters of derivatives 1A-1C is same on BN-nanotube (9, 9) surface. Results show that, theoretical and experimental trends of antiviral activity of derivatives 1A-1C were similar.
